# Dose–response relationship between dietary inflammatory index and diabetic kidney disease in US adults

**DOI:** 10.1017/S1368980022001653

**Published:** 2023-03

**Authors:** Yong-Jun Wang, Yang Du, Guo-Qiang Chen, Zhen-Qian Cheng, Xue-Mei Liu, Ying Lian

**Affiliations:** 1 Department of Health Management & Engineering Laboratory for Health Management, The First Affiliated Hospital of Shandong First Medical University & Shandong Provincial Qianfoshan Hospital, Jingshi Road 16766, Jinan 250014, People’s Republic of China; 2 Department of Clinical Nutrition, Shandong Provincial Qianfoshan Hospital & The First Affiliated Hospital of Shandong First Medical University, Jinan, People’s Republic of China; 3 Department of Medical Record Management and Statistics, Shandong Provincial Qianfoshan Hospital & The First Affiliated Hospital of Shandong First Medical University, Jinan, People’s Republic of China

**Keywords:** Dietary inflammatory index, Diabetic kidney disease, Dose–response

## Abstract

**Objective:**

The impact of the dietary potential inflammatory effect on diabetic kidney disease (DKD) has not been adequately investigated. The present study aimed to explore the association between dietary inflammatory index (DII) and DKD in US adults.

**Design:**

This is a cross-sectional study.

**Setting:**

Data from the National Health and Nutrition Examination Survey (2007–2016) were used. DII was calculated from 24-h dietary recall interviews. DKD was defined as diabetes with albuminuria, impaired glomerular filtration rate or both. Logistic regression and restricted cubic spline models were adopted to evaluate the associations.

**Participants:**

Data from the National Health and Nutrition Examination Survey (2007–2016) were used, which can provide the information of participants.

**Results:**

Four thousand two-hundred and sixty-four participants were included in this study. The adjusted OR of DKD was 1·04 (95 % CI 0·81, 1·36) for quartile 2, 1·24 (95 % CI 0·97, 1·59) for quartile 3 and 1·64 (95 % CI 1·24, 2·17) for quartile 4, respectively, compared with the quartile 1 of DII. A linear dose–response pattern was observed between DII and DKD (*P*_nonlinearity_ = 0·73). In the stratified analyses, the OR for quartile 4 of DII were significant among adults with higher educational level (OR 1·83, 95 % CI 1·26, 2·66) and overweight or obese participants (OR 1·67, 95 % CI 1·23, 2·28), but not among the corresponding another subgroup. The interaction effects between DII and stratified factors on DKD were not statistically significant (all *P* values for interactions were >0·05).

**Conclusions:**

Our findings suggest that a pro-inflammatory diet, shown by a higher DII score, is associated with increased odd of DKD.

Diabetic kidney disease (DKD) is the leading cause of chronic kidney disease and end-stage renal disease, accounting for an estimated 50 % of all end-stage renal disease cases globally^(1,2)^. It has been confirmed that DKD, as one of the most common microvascular complications of diabetes, is associated with considerable morbidity and mortality^(3)^. In addition, the progression of DKD to end-stage renal failure frequently requires renal replacement therapy, which carries with substantial health care costs^(4)^. With the rising incidence of diabetes mellitus, threat from DKD will possibly be exacerbated^(5)^. Therefore, it is imperative to uncover additional modifiable factors in addition to the classic factors for DKD, which would be helpful in developing targeted prevention strategies.

The pathophysiological changes of DKD are likely attributable to the metabolic and haemodynamic abnormalities^(6)^; however, the exact underlying mechanisms are complex and may involve multiple pathways. DKD is regarded as a metabolic-driven immunological disease^(7)^. Existing evidence has suggested associating DKD risk with inflammation, indicating that both systemic and local renal inflammation play crucial roles in the development of DKD^(8)^. Diet, as one of modifiable lifestyles, should not be neglected as a potential source of inflammation because of the property of specific nutrient. Existing studies indicated that some anti-inflammatory nutrients, for example, fibre, vitamin D and *n*-3 PUFA, are associated with lower serum levels of inflammatory biomarkers and lower risk of albuminuria and then slower kidney function decline^(9–11)^. Of note, current clinical guidelines recommend a comprehensive approach examining the effects of overall diet rather than solely looking at individual nutrient, considering that it allows for easier translation into practical dietary advice^(12)^. Therefore, assessing the overall inflammatory potential of diet and understanding the relation of diet-induced inflammation with DKD risk are important, as it may offer a unique perspective to develop strategy to alter dietary habits and harness the onset and progression of DKD.

The dietary inflammatory index (DII) represents a new tool to measure the dietary inflammatory potential^(13)^, which has been proved to be associated with inflammation^(14,15)^. Accumulating evidence has identified DII to be a potential risk factor for various diseases, such as cancers^(16)^ and diabetes^(17)^, which are risk factors for DKD. However, fewer studied have investigated the association between dietary inflammatory potential and kidney health, and some limitations have been proposed. For example, previous studies only conducted in specific population (e.g. specific age groups)^(18)^. Yet, no study has investigated the effect of the dietary inflammatory potential on DKD in a nationally representative sample, and the pattern of dose–response associations remains to be explored.

Because of lack of exhaustive estimate on the relationship, it is imperative to undertake an updated, comprehensive research to bridge the knowledge gap. Accordingly, the present study using data from the National Health and Nutrition Examination Survey (NHANES) aimed to assess if the DII is associated with DKD and to determine if the association presents in a dose–response manner.

## Materials and methods

### Study sample

The NHANES is a nationally representative survey conducted in the US population, aimed to assess health and nutrition status^(19)^. The periodic surveys, in which a stratified multistage probability sampling method was used, included data regarding demographics, socio-economics, lifestyle habits and laboratory tests. The details of NHANES are available elsewhere^(20)^. The written informed consent was obtained from all participants^(21)^.

**Fig. 1:**
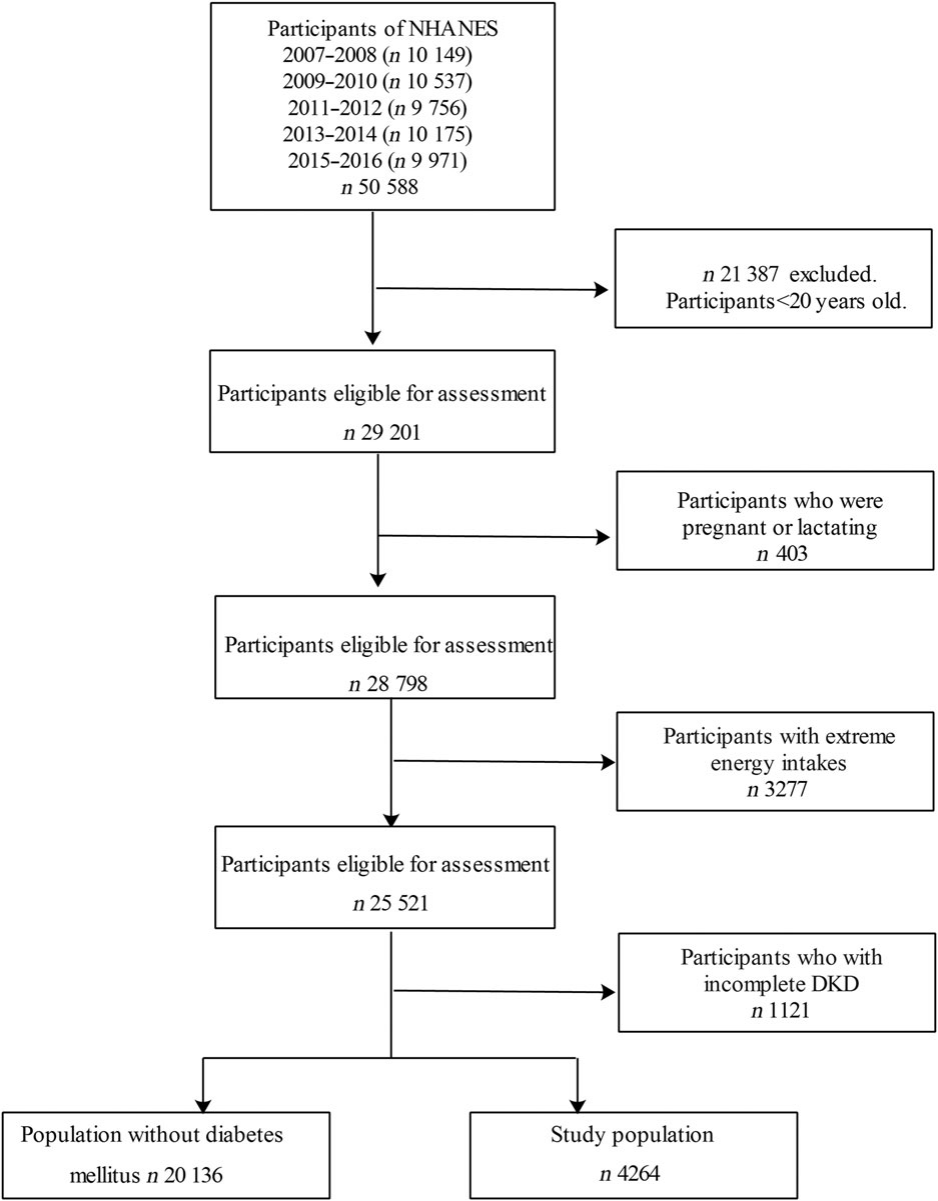
Flow chart of study sample. NHANES, National Health and Nutrition Examination Survey; DKD, diabetic kidney disease

Data from five NHANES cycles (2007–2016) were incorporated in the present study. Participants aged 20 and under were excluded (*n* 21 387). We excluded participants who were pregnant or lactating (*n* 403). According to previous studies, participants with unusual energy intakes of less than 2092 kJ/d (500 kcal/d) or above 20 920 kJ/d (5000 kcal/d) in females and less than 2092 kJ/d (500 kcal/d) or above 33 472 kJ/d (8000 kcal/d) in males (*n* 3277) were excluded^(22)^. Other participants were excluded for missing values of DKD (*n* 1121). Remaining 4264 participants with diabetes were included in the analyses. Figure 1 presents the flow chart of study sample.

### Definition of diabetic kidney disease

DKD was defined as diabetes with the presence of albuminuria, impaired glomerular filtration rate (GFR) or both^(23)^. Diabetes was defined as (1) a self-reported previous diagnosis by health care professionals, (2) fasting plasma glucose level of 7·0 mmol/l or higher, (3) HbA1c concentration of 6·5 % or higher or (4) taking glucose-lowering medications. Albuminuria was defined as the ratio of urine albumin to creatinine (ACR) of 30 mg/g or higher. Chronic Kidney Disease Epidemiology Collaboration equation was used to estimate the GFR. GFR less than 60 ml/min per 1·73 m^2^ was defined as impaired GFR.

### Assessment of dietary inflammatory index

The DII was computed based on the dietary intake data gathered by day 24-h dietary recalls. We calculated the DII score for twenty-seven food parameters available. Table 1 presents the mean intakes of the included food parameters. The calculation of DII was as follows: first a *Z*-score was calculated by subtracting the ‘standard global mean’ from the reported amount for each food parameter, which were then divided by the sd. The *Z* value was converted to a percentile score and transformed to a centred score. The corresponding inflammatory effect score multiplied by the above derived values to produce the DII score. A lower DII score represents a more anti-inflammatory diet, whereas a higher DII score represents a more pro-inflammatory diet. In our analyses, DII score varied between −4·73 and 4·55 and categorised into quartiles: quartile 1 (Q1: −4·73, −0·52), quartile 2 (Q2: −0·51, 1·01), quartile 3 (Q3: 1·02, 2·30) and quartile 4 (Q4: 2·31, 4·55).

**Table 1:** Food parameters included in the dietary inflammatory index

Food parameter	Mean intake
Carbohydrate (g)	218·70
Energy (kcal)	1832·32
Protein (g)	75·97
Fat (g)	71·56
Fibre (g)	16·25
Cholesterol (mg)	291·17
SFA (g)	22·92
MUFA (g)	25·81
PUFA (g)	16·38
*β*-carotene (μg)	2150·31
Vitamins A (RE)	607·49
Vitamins B_1_ (mg)	1·48
Vitamins B_2_ (mg)	1·91
Vitamins B_6_ (mg)	1·88
Vitamins B_12_ (μg)	4·91
Vitamins C (mg)	77·07
Vitamins D (μg)	4·50
Vitamins E (mg)	7·32
Folic acid (μg)	363·36
Fe (mg)	13·86
Mg (mg)	271·58
Zn (mg)	10·43
Se (μg)	106·15
*n*-3 PUFA (g)	1·65
*n*-6 PUFA (g)	14·55
Alcohol (g)	4·63
Caffeine (g)	0·14

### Covariates

The following potential covariates included in the analyses were selected based on literature review and availability in our data set: age, sex, race (non-Hispanic White, non-Hispanic Black, Mexican American, other Hispanic and other race), educational level (below high school, high school and above), marriage status (married/living with partner, widowed/divorced/separated/never married), family poverty income ratio, smoking status (never, current and former), drinking status (no, yes), physical activity level (low, moderate and high), BMI and hypertension. The physical activity was assessed using the Global Physical Activity Questionnaire. BMI was calculated as weight in kilograms divided by height in meters squared. Participants rested quietly in a sitting position for 5 min, and three consecutive blood pressure readings were obtained and averaged. Hypertension was defined as the average systolic blood pressure ≥ 140 mmHg or diastolic blood pressure ≥ 90 mmHg or use of anti-hypertensive medication. All above related information was gathered through standardised questionnaire, physical examination and laboratory tests.

### Statistical analysis

Sample characteristics were compared using the ANOVA for continuous variables, and the *χ* ^2^ tests for categorical variables. Binary logistic regression models were conducted to examine the association between DII and DKD with OR and 95 % CI, in which the Q1 of DII was the reference category. We firstly adjusted for age, and sex, then for race, educational level, marriage status, family poverty income ratio, smoking status, drinking status, physical activity level, hypertension and BMI in the multivariable-adjusted model. Furthermore, restricted cubic spline models with knots at the 5th, 35th, 65th and 95th percentiles were performed to explore the shape of dose–response relationship between DII and DKD adjusted for all above covariates^(24)^. In addition, stratified analyses were carried out by socio-demographic and clinical characteristics including sex, age (middle-aged adults: < 60 years old, older adults: ≥60 years old), educational level, BMI (underweight/normal: ≤ 24·9 kg/m^2^, overweight/obese: >25 kg/m^2^) and status of hypertension. Stata 15.0 software (StataCorp., LP) was used in all analyses.

## Results

Among 4264 participants, the weighted proportion of DKD was 36·2 %. The characteristics of our sample across quartiles of DII score are shown in Table 2. Participants in the Q4 of DII score were older, more likely to be female, single, have lower educational level, less physical activity, less household income, higher BMI, current smoking and drinking. The prevalence of hypertension, DKD, impaired GFR and albuminuria was higher among participants with the most pro-inflammatory diet.

**Table 2:** Characteristics of study sample according to DII quartiles

Characteristics	Q1 of DII (−4·73, −0·52)		Q 2 of DII (−0·51, 1·01)		Q3 of DII (1·02, 2·30)		Q4 of DII (2·31, 4·55)	
	Mean	sd	Mean	sd	Mean	sd	Mean	sd
Age (years)	59·99	13·13	60·79	13·53	60·54	13·42	61·86	13·62
	*n*	%	*n*	%	*n*	%	*n*	%
Sex								
Male	711	66·70	614	57·60	491	46·06	425	39·87
Female	355	33·30	452	42·40	575	53·94	641	60·13
Race								
Non-Hispanic White	397	37·24	399	37·43	348	32·65	362	33·96
Non-Hispanic Black	224	21·01	257	24·11	301	28·24	311	29·17
Mexican American	222	20·83	204	19·14	186	17·45	188	17·64
Other Hispanic	104	9·76	120	11·26	147	13·79	130	12·20
Other race	119	11·16	86	8·07	84	7·88	75	7·04
Educational level								
Below high school	274	25·70	344	32·27	419	39·30	490	45·97
High school and above	792	74·30	722	67·73	647	60·70	576	54·03
Marriage status								
Married/living with partner	705	66·13	646	60·60	601	56·38	582	54·60
Widowed/divorced/separated/never married	361	33·87	420	39·40	465	43·62	484	45·40
Family poverty income ratio								
≤1	207	19·41	219	20·50	297	27·84	341	31·99
1–1·84	245	22·97	290	27·22	287	26·90	326	30·54
≥1·85	614	57·62	557	52·28	482	45·26	399	37·47
Smoking status								
Never	549	51·50	511	47·94	530	49·72	527	49·44
Current	127	11·91	163	15·29	193	18·10	222	20·83
Former	390	36·59	392	36·77	343	32·18	317	29·74
Drinking status								
No	760	71·25	708	66·40	676	63·37	607	56·93
Yes	306	28·75	358	33·60	390	36·63	459	43·07
Physical activity level								
Low	628	58·89	675	63·32	718	67·35	748	70·16
Moderate	282	26·43	257	24·11	234	21·95	219	20·59
High	156	14·68	134	12·57	114	10·70	99	9·25
Hypertension	315	29·55	340	31·91	354	33·24	376	35·26
	Mean	sd	Mean	sd	Mean	sd	Mean	sd
BMI (kg/m^2^)	32·30	7·43	32·54	7·32	32·63	7·49	32·64	7·31
	*n*	%	*n*	%	*n*	%	*n*	%
DKD	380	35·65	404	37·90	449	42·12	504	47·28
Impaired GFR	165	15·46	194	18·24	233	21·90	259	24·30
Albuminuria	282	26·48	300	28·15	345	32·39	384	35·98

DII, dietary inflammatory index; DKD, diabetic kidney disease; GFR, glomerular filtration rate.

Table 3 shows the weighted associations between DII with DKD, impaired GFR and albuminuria. In the multivariable-adjusted model, the OR of DKD was 1·04 (95 % CI 0·81, 1·36) for the Q2, 1·24 (95 % CI 0·97, 1·59) for the Q3 and 1·64 (95 % CI 1·24, 2·17) for the Q4 compared with Q1 of DII score. Similar figures were 1·16 (95 % CI 0·81, 1·68), 1·35 (95 % CI 0·97, 1·88) and 1·57 (95 % CI 1·10, 2·26) for impaired GFR, and 1·00 (95 % CI 0·75, 1·33), 1·28 (95 % CI 0·94, 1·72) and 1·56 (95 % CI 1·14, 2·12) for albuminuria.

**Table 3:** Weighted OR (95 % CI) of the association between DII and DKD

Characteristics	Q1 of DII	Q2 of DII		Q3 of DII		Q4 of DII	
		OR	95 % CI	OR	95 % CI	OR	95 % CI
DKD							
Model 1*	1	1·13	0·91, 1·40	1·40	1·14, 1·72	1·91	1·57, 2·32
Model 2†	1	1·04	0·81, 1·36	1·24	0·97, 1·59	1·64	1·24, 2·17
Impaired GFR							
Model 1*	1	1·29	0·96, 1·73	1·57	1·22, 2·02	1·98	1·52, 2·57
Model 2†	1	1·16	0·81, 1·68	1·35	0·97, 1·88	1·57	1·10, 2·26
Albuminuria							
Model 1*	1	1·07	0·85, 1·36	1·41	1·10, 1·81	1·88	1·48, 2·39
Model 2†	1	1·00	0·75, 1·33	1·28	0·94, 1·72	1·56	1·14, 2·12

DII, dietary inflammatory index; DKD, diabetic kidney disease; GFR, glomerular filtration rate.

* Model 1 adjusted for age and sex.

† Model 2 adjusted for age, sex, race, educational level, marriage status, family poverty income ratio, smoking status, drinking status, physical activity level, hypertension and BMI.

**Fig. 2:**
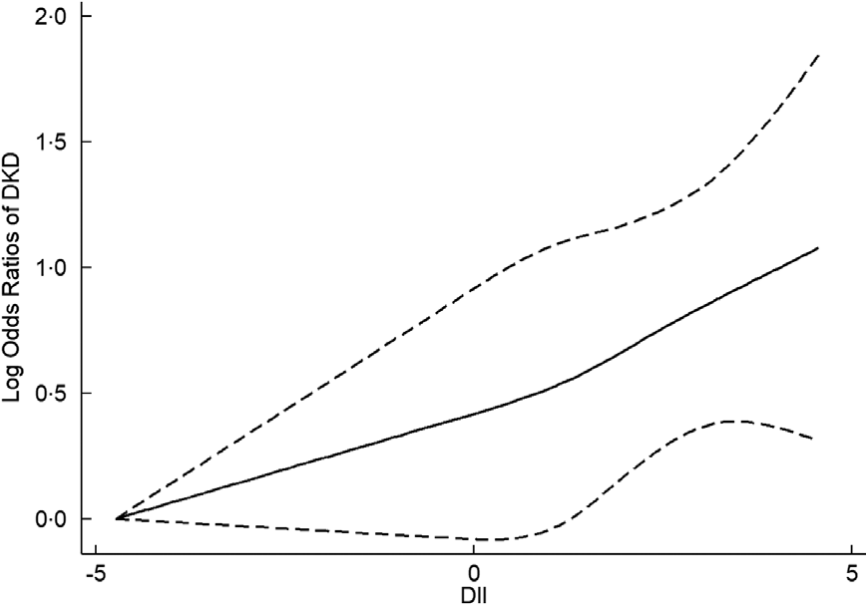
The dose–response relationship between dietary inflammatory index (DII) and diabetic kidney disease (DKD)

In the cubic spline model, a linear dose–response relationship was found between DII and DKD (*P*_nonlinearity_ = 0·73). The adjusted OR for per unit increasing of DII was 1·13 (95 % CI 1·07, 1·19) for DKD. The dose–response relationship between DII and DKD is presented in Fig. 2.

In the stratified analyses, the association of DKD for the Q4 of DII was statistically significant among adults with higher educational level, with OR 1·83 (95 % CI 1·26, 2·66), but not among adults with lower educational level. The association for the Q4 was statistically significant among overweight or obese participants, with OR 1·67 (95 % CI 1·23, 2·28), but not among underweight/normal participants. The interaction effects between DII and stratified factors on DKD were not statistically significant (all *P* values for interactions were >0·05). The associations of DII with DKD in stratified analyses are shown in Table 4.

**Table 4:** The weighted OR (95 % CI) of the association between DII and DKD in stratified analyses

Characteristics	Q1 of DII	Q2 of DII		Q3 of DII		Q4 of DII		*P*_for interaction_
		OR	95 % CI	OR	95 % CI	OR	95 % CI	
Sex								0·73
Female	1	0·89	0·62, 1·31	1·20	0·82, 1·77	1·72	1·13, 2·62	
Male	1	1·49	1·07, 2·09	1·46	1·01, 2·13	1·71	1·20, 2·43	
Age								0·12
Middle-aged adults	1	0·97	0·67, 1·42	1·21	0·81,1·80	1·59	1·01, 2·51	
Older adults	1	1·23	0·89, 1·69	1·43	1·04,1·97	1·89	1·40, 2·56	
Educational level								0·23
Below high school	1	1·02	0·62, 1·70	1·15	0·76, 1·75	1·43	0·91, 2·23	
High school and above	1	0·96	0·69, 1·33	1·22	0·90, 1·64	1·83	1·26, 2·66	
BMI								0·83
Underweight/normal	1	0·39	0·16, 1·00	1·04	0·44, 2·47	1·39	0·67, 2·90	
Overweight/obese	1	1·15	0·89, 1·49	1·26	0·98, 1·62	1·67	1·23, 2·28	
Status of hypertension								0·36
Hypertension	1	0·98	0·63, 1·53	1·18	0·77, 1·82	1·97	1·29, 3·02	
Non-hypertension	1	1·21	0·91, 1·94	1·43	1·07, 1·90	1·65	1·19, 2·29	

DII, dietary inflammatory index; DKD, diabetic kidney disease.

Models adjusted for age, sex, race, educational level, marriage status, family poverty income ratio, smoking status, drinking status, physical activity level, hypertension and BMI.

## Discussion

In the present study, we explored the association between DII and DKD using a nationally representative sample of US adults. A more pro-inflammatory diet, as estimated by a higher DII score, is associated with increased odd of DKD. Particularly important, the association presents in a linear dose–response manner.

The findings were consistent with previous studies on the association between dietary patterns and kidney function^(25–27)^. For example, a prospective analysis in the Nurses’ Health study found that adherence to the pro-inflammatory Western-style diet, compared with a healthier diet, was associated with an increased risk of GFR decline, with the associations no variation by diabetes status^(28)^. Conversely, the higher alternative healthy eating index, a measure of diet quality negatively correlated with DII^(29)^, was associated with a lower odd of albuminuria among population with diabetes^(30)^. Similarly, a multi-ethnic study also showed that a diet rich in fruit and wholegrains, with presumed anti-inflammatory properties, was associated with lower odds of micro-albuminuria in individuals with diabetes^(31)^. Although each dietary index represents a unique combination of dietary nutrients, to some extent, they share considerable similarities and have been significantly associated with inflammatory markers^(32)^. Notably, DII is designed to assess the dietary inflammatory potential, which represents the inflammatory mechanism underlying the diet–health link. Importantly, it is worth emphasising that our findings make a significant addition to literature by demonstrating association between DII and DKD among a nationally representative sample of population. The observed associations emphasise the potential of avoiding pro-inflammatory diet in DKD prevention.

In our study, subgroup analyses stratified by potential factors were established, followed by interaction terms to test the heterogenicity among different subgroups. The interaction between DII and stratified factors on DKD was not statistically significant, which ensures the reliability of the conclusion. This is in line with previous studies that identified similar associations between the Dietary Approaches to Stop Hypertension diet and kidney disease by sex, race and education level^(33)^. In contract, a study identified the statistically significant interaction between BMI status and alternative healthy eating index on end-stage kidney disease risk^(25)^. And, another study showed that the Dietary Approaches to Stop Hypertension diet was associated with rapid GFR decline among participants with hypertension but not among those without hypertension^(34)^. Further research is necessary to replicate these findings from stratified analyses.

The cubic spline analysis further visualised the association between dietary inflammatory potential and DKD development. A cohort study of women aged 70 years indicated that there was a linear association between DII and the baseline renal function, renal function trajectory, suggesting that one-unit higher DII score was associated with a 0·55 ml/min per 1·73 m^2^ lower GFR at baseline and a 0·06 ml/min per 1·73 m^2^ greater annual decline in GFR over 10 years^(35)^. Another study conducted among older adults indicated that an increment of 1 sd in DII was associated with lower GFR, with a *β* of −1·8 % (95 % CI −2·7 %, −0·9 %)^(18)^. Similarly, a linear dose–response pattern was found for the association between DII and DKD in the present study, in which 1-unit increasing of DII was associated with a 13 % higher odd of DKD. Previous studies have provided some supporting evidence for the benefits of maintaining an anti-inflammatory diet^(36–38)^. It is encouraging of the observed association that avoiding a pro-inflammatory diet could serve to be an additional, non-pharmacologic means for prevention of DKD. In future, more research is required to develop evidence-based preventive models, whether lowering intake of inflammation-promoting diet can translate to reducing the development of DKD.

Several possible mechanisms may explain the link between DII and DKD. First, a pro-inflammatory diet is positively associated with elevated inflammatory levels such as leucocyte counts^(39)^. Although the metabolic disorder is historically considered as the pathogenesis of DKD, recent studies have established that the inflammatory responses also play a central role in progression of DKD. It has been shown that inflammation together with neutrophil–endothelium interactions could contribute to the pathogenesis of kidney injury, potentially leading to chronically impaired kidney function. Second, the dietary inflammatory potential is well recognised to regulate oxidative processes^(40)^. It is reported that the oxidant–antioxidant imbalance plays an important pathogenic role in the development of diabetic complications, including diabetic nephropathy^(41,42)^. Furthermore, the gut–kidney axis may represent a potential pathway underlying the inflammatory diet–DKD link^(7)^. Clinical trials have further shown the mechanistic involvement of gut microbiota in the pathophysiology of DKD by proving that gut microbiota can potentially trigger immune, metabolic and fibrotic pathways, which perpetuate the progression of renal pathology^(43,44)^. As has been reported that specific pro-inflammatory nutrients included in the calculation of DII serve to be key determinants of the modulating gut microbiota composition and activity^(45,46)^.

One of the strengths is that this is the first study to explore the associations between the dietary inflammatory potential and DKD in a nationally representative sample of population. Second, data of NHANES are from a nationally representative sample of the USA, which enables the observed associations to be generalised. Third, the cubic spline analysis in our study, which characterises a dose–response association between a continuous exposure and an outcome, can help to clarify how the odd of DKD changes along with dietary inflammatory potential increasing. There are also some limitations in our study. First, our study is the cross-sectional design, which does not allow for inferences about causality. Second, as shown in online supplementary material, Supplemental Table 1, the proportion of excluded participants was relatively high, because of extreme energy intake, which might bring about bias in estimating the associations. Finally, the dietary data were from 24 h dietary recall interviews, and there might be ineluctable recall bias. Thus, well-designed longitudinal studies incorporating accurate assessment of measures are needed to fully clarify the causal relationship.

Our findings have public health and clinical significance, which is potentially important for not only the prevention but also the management of DKD. From the public health perspective, although this association was not causally shown, it may be legitimate to advise individuals to adhere to an anti-inflammatory diet to reduce their risk for kidney disease complications. Evidence-based public health education and publicity should be strengthened in an even broader segment of US population to raise awareness of altering dietary habits and promote an anti-inflammatory diet for DKD prevention. In addition, from the clinical perspective, future research should aim to evaluate the dietary inflammatory potential, develop nutritional protocol and consider incorporating it in dietary guidelines of managing DKD.

## Conclusion

In conclusion, a more pro-inflammatory diet, as estimated by the higher DII score, was significantly associated with higher odd of DKD. Our findings emphasised the importance of developing novel nutritional approaches to prevent and manage DKD. Further clinical trials are required to strengthen the evidence of the associations between DII and DKD.

## Supplementary material

For supplementary material accompanying this paper visit https://doi.org/10.1017/S1368980022001653. click here to view supplementary material
