# Supplementary material for: Dose–response relationship between dietary inflammatory index and diabetic kidney disease in US adults
Source: Public Health Nutr. 2022 Aug 9;26(3):611–9. doi: 10.1017/S1368980022001653 (PMC9989711; doi:10.1017/S1368980022001653)
Supplement: Supplementary file 1 [file S1368980022001653sup001.docx]

Supplementary Table 1 the characteristics of participants with plausible and implausible energy intakes

| Characteristics | Participants with plausible calorie intake (n=4264) | Participants with implausible calorie intake (n=3277) |
| --- | --- | --- |
| Age (years) | 60.79±13.44 | 51.09±18.71 |
| Sex (%) |  |  |
| Male | 2241 (52.56) | 1526 (46.57) |
| Female | 2023 (47.44) | 1751 (53.43) |
| Race (%) |  |  |
| Non-Hispanic White | 1506 (35.32) | 1031 (31.46) |
| Non-Hispanic Black | 1093 (25.63) | 724 (22.09) |
| Mexican-American | 800 (18.76) | 484 (14.5977) |
| Other Hispanic | 501 (11.75) | 399 (12.18) |
| Other race | 364 (8.54) | 639 (19.50) |
| Educational level (%) |  |  |
| Below high school | 1527 (35.81) | 1062 (32.41) |
| High school and above | 2737 (64.19) | 2215 (67.59) |
| Marriage status (%) |  |  |
| Married/Living with partner | 2534 (59.43) | 1779 (54.29) |
| Widowed/Divorced/Separated/Never married | 1730 (40.57) | 1498 (45.71) |
| Family poverty income ratio (%) |  |  |
| ≤1 | 1064 (24.95) | 940 (28.68) |
| 1-1.84 | 1148 (26.92) | 832 (25.38) |
| ≥1.85 | 2052 (48.12) | 1505 (45.94) |
| Smoking status (%) |  |  |
| Never | 2117 (49.65) | 1889 (57.64) |
| Current | 705 (16.53) | 697 (21.27) |
| Former | 1442 (33.82) | 691 (21.09) |
| Drinking status (%) |  |  |
| No | 2751 (64.52) | 1917 (58.51) |
| Yes | 1513 (35.48) | 1360 (41.49) |
| Physical activity level (%) |  |  |
| Low | 2769 (64.94) | 1854 (56.57) |
| Moderate | 992 (23.26) | 886 (27.04) |
| High | 503 (11.80) | 537 (16.39) |
